# Using ChatGPT to evaluate the integration status of wetland conservation in local plans

**DOI:** 10.1016/j.isci.2026.118098

**Published:** 2026-11-03

**Authors:** Haishan Lin, Beichen Zhang, Xiang Li, Yang Zhang, Xuguang Tang, Ligang Zhang

**Affiliations:** 1 Zhejiang Provincial Key Laboratory of Wetland Intelligent Monitoring and Ecological Restoration, Institute of Remote Sensing and Earth Sciences, Hangzhou Normal University, Hangzhou 311121, China; 2 Earth and Environmental Sciences Area, Lawrence Berkeley National Laboratory, Berkeley, CA 94720, USA; 3 Zhejiang Institute of Hydraulics & Estuary, Hangzhou 310020, China

**Keywords:** plan evaluation, language models, ChatGPT, local comprehensive plan, Nebraska

## Abstract

Plan evaluation supports the development of higher quality plans. We examined whether language models could assist evaluation of wetland conservation in local comprehensive plans through prompt optimization. Using GPT-4o mini as both optimizer and evaluator, we developed prompts on five Nebraska county plans and compared basic and optimized prompts for GPT-4o and GPT-4o mini on twenty held-out plans across 56 indicators. Mean county-level agreement with human reference scores increased from 47.69% to 62.20% for GPT-4o and from 43.35% to 58.75% for GPT-4o mini under ternary scoring. This approach may enhance the reliability of language models for assessing planning quality and support planners in evaluating wetland conservation in local plans.

## Highlights

• Optimized prompts raised ternary model-human agreement by about 15 percentage points
• Post-hoc binary scoring showed higher model-human agreement than ternary scoring
• LLMs struggled to distinguish partial from full wetland conservation integration
• Human verification remains necessary for LLM-assisted plan evaluation

## Graphical abstract

**Figure:**
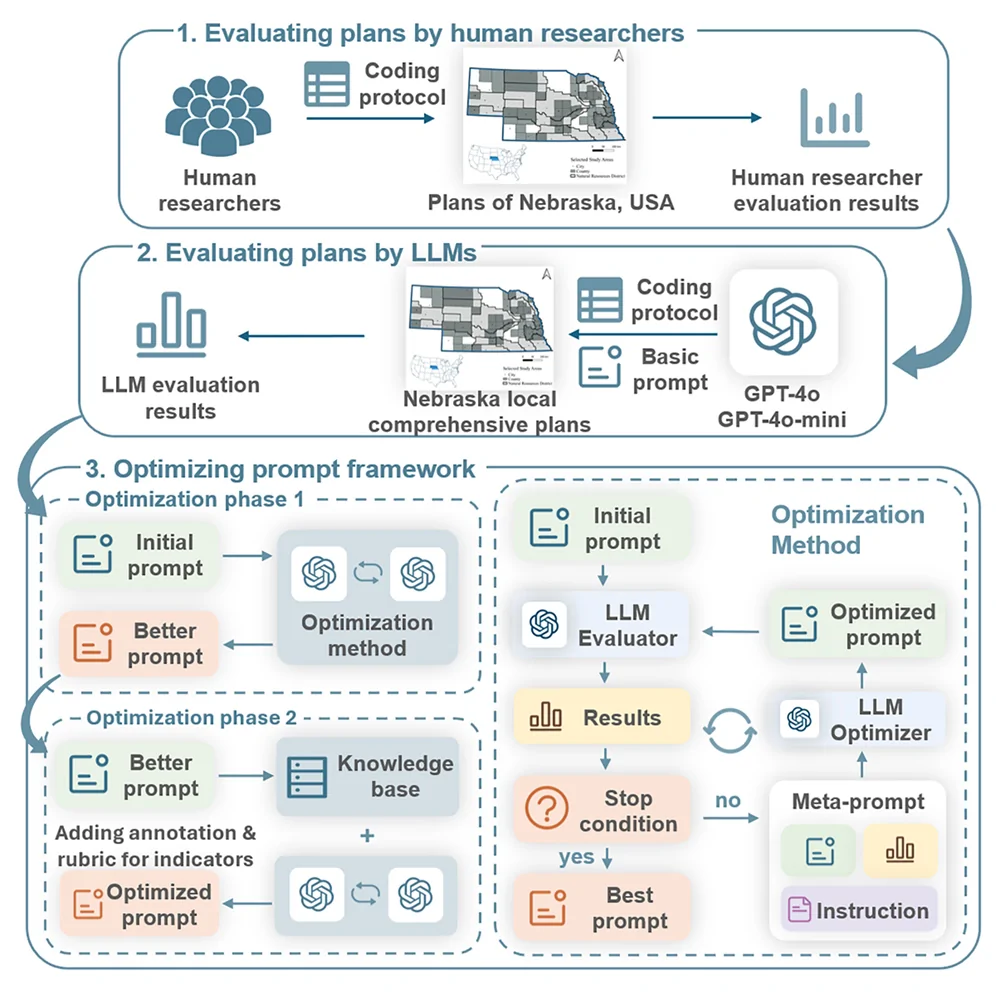

## Teaser

Applied sciences; Artificial intelligence

## Introduction

Urban planning plays a vital role in steering and regulating urban development and is often seen as the cornerstone of the field. A planning document typically serves as a long-term policy instrument that manages and guides local development.^1^ It functions as a practical, goal-oriented, and policy-based framework for translating development visions of society into practical implementation. As a crucial guiding document, a plan is typically developed through meticulous field research, along with the verification and incorporation of public feedback.^2^^,^^3^^,^^4^ This process renders the plan a comprehensive repository of local knowledge and information. In addition to guiding and regulating urban development, plans have been described by Neuman^4^; Ryan^5^ as “the major printed currency of the planning profession”. Reading and analyzing planning documents have always been central to the planning profession. Such analysis helps practitioners acquire new insights for professional development, enables researchers to advance theoretical frameworks, and enhances the effective implementation of plans.^5^^,^^6^^,^^7^ Over the last three decades, numerous studies have evaluated a wide range of plans, including those addressing climate change, local development, and natural hazards.^7^^,^^8^^,^^9^ Plan quality evaluation research is widely recognized as a powerful learning tool for improving the quality of current and future plans.^8^^,^^10^ However, relatively few studies have focused on how to increase the efficiency of plan evaluation. With the rapid development of AI technologies, particularly large language models (LLMs) such as ChatGPT, we aim to explore how such tools can be applied to the field of plan evaluation.

The recent breakthrough progress in LLMs has sparked widespread interest and research activity across academic and industrial sectors globally.^11^^,^^12^^,^^13^ Representative LLMs, including GPT-3,^14^ GPT-4,^15^ BERT,^16^ Llama-2,^17^ and Claude,^18^ have demonstrated state-of-the-art performance across diverse natural language processing (NLP) tasks. LLMs apply a two-stage paradigm: unsupervised pretraining on unannotated corpora, followed by supervised fine-tuning for downstream tasks. This framework enables versatile capabilities across diverse NLP applications and effectively addresses the limitations of earlier models constrained to specific tasks and hampered by the scarcity of task-specific labeled datasets.^19^ Due to their exceptional cross-domain adaptability, LLMs have become the primary solution for diverse users addressing NLP challenges, with specialized applications proliferating across professional fields. LLMs have demonstrated formidable application potential across multiple domains. In specialized fields with stringent professional requirements, such as medicine,^20^^,^^21^ agriculture,^22^^,^^23^ finance,^24^^,^^25^^,^^26^ and legal practice, LLMs have already been implemented to support decision-making processes, conduct report generation, and perform risk assessments.

Evaluating spatial policies in planning documents differs fundamentally from text evaluation in corporate or medical domains. Comprehensive plans are inherently spatially referenced documents, organized around geographic hierarchies and land-use classifications. This spatial taxonomy challenges language models in two ways. First, a policy’s evaluative weight may shift with its geographic referent. Second, logical coherence must be assessed across sections linked spatially rather than semantically. Moreover, planning documents are structurally heterogeneous, and evaluation often requires retrieving implicit evidence scattered across chapters.^8^ Corporate reports and clinical notes, by contrast, follow standardized formats with more explicit evaluation criteria. These differences explain why general-purpose LLMs struggle with plan evaluation and underscore the need for domain-specific adaptation. In the domain of urban planning, however, the adoption of LLMs has largely concentrated on spatial analysis and risk modeling, while their application to plan quality evaluation remains notably underexplored. This gap is not merely one of application but of methodology: to date, no systematic framework exists for adapting general-purpose LLMs to the specialized linguistic and evaluative demands of planning documents.

LLMs still face several challenges in performing planning evaluation tasks. First, plan evaluation requires coders to possess deep domain expertise, presenting high professional barriers for both humans and LLMs. Local comprehensive plans contain abundant domain-specific terminologies, which may cause hallucinations in LLMs during input comprehension and processing,^27^^,^^28^ thereby undermining the reliability of results. These hallucinations could manifest as fabricated content or factual inaccuracies in LLM responses.^27^^,^^29^ Furthermore, linguistic and terminology variations across different regions and languages exacerbate LLMs’ biases in domain-specific NLP tasks.^30^^,^^31^^,^^32^^,^^33^ Second, the sheer volume and extensive spatiotemporal scope of plan documents present fundamental challenges for processing. These difficulties are further compounded by the inherent input length constraints of LLMs. Earlier research primarily focused on traditional NLP techniques, such as machine learning-based classifiers, clustering, and topic modeling, to assist planners in document analysis.^34^ Fu et al. were the first study to apply GPT-3.5 to planning quality evaluation.^8^ However, the work was limited to a direct zero-shot evaluation approach and revealed a critical *concept alignment* problem: generic LLMs frequently misinterpret domain-specific planning terminology, and simple prompting fails to resolve this. The study concluded that while LLMs hold promise, substantial methodological advancement is required to achieve acceptable reliability. Overall, the application of LLMs in plan evaluation remains in its early stage.

In recent research, the application of LLMs for specific tasks typically takes two primary forms. The first entails tailoring partial or full parameters of the model to a task via fine-tuning. Conversely, the second leverages zero-shot or few-shot prompts to process tasks directly while freezing all model parameters. To achieve accurate results on specialized tasks, effective prompt engineering is essential. This involves crafting instructions that guide models toward completing the task and generating the desired outputs.^35^^,^^36^^,^^37^ PromptRobust^38^ demonstrates the notable sensitivity of LLMs to adversarial prompts, showing that even subtle semantic nuances between prompts can trigger performance fluctuations. Inspired by these findings, we investigate whether optimized prompt engineering can enhance the accuracy of LLMs in planning quality evaluation.

To address the investigation of the possibility of using LLMs in plan evaluation, three specific questions are addressed in this research:

(1) Can LLMs conduct plan evaluation using optimized prompts?
(2) How can prompt systems for LLMs be optimized to improve plan evaluation performance on integrating wetland conservation into local comprehensive plans?
(3) Where do LLMs perform well in plan evaluation, and where do they struggle?

## Results

### Overview of plan evaluation by LLM

To address the three research questions, we systematically evaluated LLM performance on 20 county-level comprehensive plans from Nebraska, focusing on the integration of wetland conservation policies. We used a 2 × 2 final-evaluation design that crossed two model labels (GPT-4o and GPT-4o mini) with two prompt conditions (basic and optimized). This design yielded four configurations (Table 1)—GPT-4o/basic, GPT-4o/optimized, GPT-4o mini/basic, and GPT-4o mini/optimized—each evaluated on the same 20 held-out county plans and 56 indicators (Table 2). Model outputs were compared with human expert consensus using percent agreement and Krippendorff’s alpha, which accounts for chance agreement. Section 5 details the prompt design, optimization protocol, and retrieval parameters. The following sections present the principal empirical findings.

**Table 1:** Final evaluation configurations

Archived model label	Final role	Prompt condition	Retrieval cap	Chunking	Temperature/API settings	Scope/runs	Run start
GPT-4o	Final evaluator	Basic	20	800 tokens; 400 overlap	temperature = 0	20 plans × 56 indicators; one full run	25 Feb 2025
GPT-4o	Final evaluator	Optimized	20	800 tokens; 400 overlap	temperature = 0	20 plans × 56 indicators; one full run	23 Feb 2025
GPT-4o mini	Final evaluator	Basic	20	800 tokens; 400 overlap	temperature = 0	20 plans × 56 indicators; one full run	27 Feb 2025
GPT-4o mini	Final evaluator	Optimized	20	800 tokens; 400 overlap	temperature = 0	20 plans × 56 indicators; one full run	1 Mar 2025

Note: All configurations are reported with temperature = 0 and a File Search retrieval cap of 20 (file_search.max_num_results = 20). Dates are run starts recorded in the original result filenames. The accompanying evaluation_configurations.json specifies each condition and its result file. Other generation settings are not explicitly overridden in the supplied scripts. Complete run-level sampling metadata are not retained in the archived outputs.

**Table 2:** Indicators used to evaluate the comprehensive plans

Category	Indicators
Factual Basis	Natural or environmental resources described (open space, green space, river corridors)
	Conservation areas mapped/described
	Environmental sensitive area described
	Wetland current status and description (historical loss/gain/degradation/challenges)
	Wetland definition
	Wetland maps and classifications
	Local wetland types/classification
	Conservation projects/programs
	Conservation prioritized in land use development
	Conservation lands identified on land use maps/plans
	Watershed management programs
	Water resources related description (e.g., quantity and quality, pollution)
	Watershed development status (ISA, stormwater)
Goals and objectives	Goals for no net loss
	Goals for anti-degradation
	Goals for increasing wetland quantity and quality
	Goals for ecosystem/biodiversity protection (Wildlife, bird, crane)
	Environmental sustainability/resilience goal
	Manmade/constructed wetland and wetland restoration
	Future land use and zoning regulations
	Zoning policies for specific areas for protection
	Low density design standard and building codes for conservation
	Regulate wetland disturbance and impact activities
	Prohibit development in environmental sensitive areas
	Subdivision regulations on open space and park land
	Policies on rural character maintaining
	Road and transportation standard adjustment for conservation
	Conservation design standards and techniques
	Water resource protection
	Environmental regulations for natural system protection
	Regulations on ecosystem and biodiversity conservation (Wildlife, bird, crane)
	Regulations for buffer zone
	Wetland/water management and restoration
	Permitted use protecting natural system
	Stormwater management
	Water related BMPs
	Local tax incentives to support conservation
	Mixed use development/Low impact development
	Development rights related strategies
	Clustered development
	Density bonuses
	Conservation easement
	Land acquisition program for conservation
	Financial/economic incentive for conservation
	Conservation education/outreach projects/programs/activities
	Natural educational opportunities
	Waterfront recreational sites for education/educational facilities
Coordination and integration	Link local approvals with 404 permit and/or 401 certifications/swampbuster/title 117
	Coordination with local/state/federal agencies
	Coordination with NGOs or environmental organizations/research institutes/participants
	Integration with other plans/policies/programs
Implementation, monitoring, and updating	Implementation timelines/trend
	Wetland-related task force/committee
	Maintenance and updating procedure and development continuum
	Plan effectiveness on conservation monitoring
	Implementation of conservation development techniques/practices

Overall, current language models remained below the 80% operational reference commonly used in plan-evaluation research under the 0–1–2 ternary scoring system (Tables 3, 4, and 5). Mean county-level agreement was 47.69% for GPT-4o/basic (95% CI: 44.43%–51.35%) and 62.20% for GPT-4o/optimized (95% CI: 58.98%–65.42%). The paired optimized-minus-basic difference for GPT-4o was 14.51 percentage points (95% CI: 11.36–17.78). For GPT-4o mini, mean county-level agreement was 43.35% under the basic prompt condition (95% CI: 38.87%–47.90%) and 58.75% under the optimized prompt condition (95% CI: 53.66%–63.57%), yielding a paired difference of 15.40 percentage points (95% CI: 9.98–20.67). These validation estimates indicate improved model-human agreement after prompt optimization, although the absolute ternary agreement levels remain insufficient for fully autonomous plan evaluation. For GPT-4o/optimized, 13 of 20 validation counties had ordinal Krippendorff’s alpha values of at least 0.33; four values ranged from 0.33 to 0.40 and nine exceeded 0.40. The 0.33–0.40 range is reported as a context-specific reference for dispersed plan-quality coding tasks.^8^^,^^39^

**Table 3:** Agreement under ternary scoring criteria (0–1–2) for the 20 counties in the validation set

Counties	4o final		4o basic		4o-mini final		4o-mini basic	
	Consistency	Alpha	Consistency	Alpha	Consistency	Alpha	Consistency	Alpha
Antelope	55.36%	0.39	47.27%	0.48	51.79%	0.33	42.86%	0.33
Buffalo	75.00%	0.46	48.21%	0.25	51.79%	0.02	39.29%	0.18
Butler	64.29%	0.35	57.14%	0.36	69.64%	0.48	35.71%	0.16
Dakota	64.29%	0.53	44.64%	0.54	62.50%	0.29	21.82%	0.14
Dawes	69.64%	0.42	62.50%	0.48	67.86%	0.38	44.64%	0.29
Furnas	51.79%	0.23	42.86%	0.20	53.57%	0.17	41.07%	0.06
Greeley	62.50%	0.25	42.86%	0.62	64.29%	0.13	33.93%	0.01
Hitchcock	60.71%	0.41	55.77%	0.33	62.50%	0.48	51.79%	0.40
Howard	65.45%	0.35	42.59%	0.42	66.07%	0.22	62.50%	0.51
Kearney	64.29%	0.28	46.43%	0.29	53.57%	0.15	34.55%	0.14
Kimball	73.21%	0.58	50.00%	0.24	75.00%	0.46	50.00%	0.31
Knox	57.14%	0.52	43.64%	0.14	41.07%	0.32	39.29%	0.17
Lincoln	57.14%	0.33	44.64%	0.08	55.36%	0.31	50.00%	0.01
Nance	64.29%	0.44	42.31%	0.36	64.29%	0.35	46.43%	0.45
Perkins	50.00%	0.21	46.43%	0.39	58.93%	0.29	32.14%	0.23
Red Willow	60.71%	0.58	35.71%	0.39	37.50%	0.28	28.57%	0.14
Seward	48.21%	0.25	41.07%	0.40	42.86%	0.22	48.21%	0.03
Valley	71.43%	0.40	62.50%	0.36	80.36%	0.17	58.93%	0.33
Wayne	58.93%	0.32	35.85%	0.20	46.43%	0.22	50.00%	0.30
York	69.64%	−0.16	60.71%	0.06	69.64%	−0.04	55.36%	0.16

**Table 4:** Agreement under ternary scoring criteria (0–1–2) for the 56 indicators in the validation set

Indicators	4o final	4o basic	4o-mini final	4o-mini basic
Wetland definition	95.00%	100.00%	100.00%	85.00%
Wetland maps and classifications	95.00%	80.00%	85.00%	75.00%
Local wetland types/classification	95.00%	85.00%	90.00%	85.00%
Goals for no net loss	95.00%	75.00%	80.00%	40.00%
Coordination with NGOs or environmental organizations/research institutes/participants	95.00%	95.00%	70.00%	70.00%
Wetland-related task force/committee	95.00%	20.00%	95.00%	95.00%
Density bonuses	90.00%	65.00%	70.00%	60.00%
Conservation easement	85.00%	35.00%	75.00%	70.00%
Link local approvals with 404 permit and/or 401 certifications/swampbuster/title 117	85.00%	45.00%	80.00%	70.00%
Future land use and zoning regulations	80.00%	25.00%	85.00%	20.00%
Land acquisition program for conservation	80.00%	60.00%	75.00%	70.00%
Plan effectiveness on conservation monitoring	80.00%	75.00%	45.00%	50.00%
Watershed management programs	75.00%	45.00%	70.00%	40.00%
Manmade/constructed wetland and wetland restoration	75.00%	30.00%	75.00%	80.00%
Zoning policies for specific areas for protection	75.00%	60.00%	85.00%	30.00%
Prohibit development in environmental sensitive areas	75.00%	10.00%	70.00%	55.00%
Regulations for buffer zone	75.00%	45.00%	65.00%	35.00%
Clustered development	75.00%	75.00%	55.00%	20.00%
Goals for anti-degradation	70.00%	70.00%	75.00%	75.00%
Goals for increasing wetland quantity and quality	70.00%	35.00%	75.00%	50.00%
Environmental regulations for natural system protection	70.00%	60.00%	65.00%	35.00%
Local tax incentives to support conservation	70.00%	50.00%	65.00%	45.00%
Development rights related strategies	70.00%	85.00%	40.00%	15.00%
Financial/economic incentive for conservation	70.00%	50.00%	60.00%	45.00%
Waterfront recreational sites for education/educational facilities	70.00%	35.00%	70.00%	25.00%
Conservation lands identified on land use maps/plans	65.00%	25.00%	50.00%	15.00%
Subdivision regulations on open space and park land	65.00%	40.00%	55.00%	65.00%
Mixed use development/Low impact development	65.00%	40.00%	55.00%	5.00%
Integration with other plans/policies/programs	65.00%	15.00%	30.00%	50.00%
Implementation of conservation development techniques/practices	65.00%	85.00%	40.00%	5.00%
Wetland current status and description (historical loss/gain/degradation/challenges)	60.00%	60.00%	35.00%	40.00%
Watershed development status (ISA, stormwater)	60.00%	55.00%	65.00%	45.00%
Environmental sustainability/resilience goal	60.00%	20.00%	60.00%	50.00%
Water related BMPs	60.00%	30.00%	60.00%	5.00%
Conservation education/outreach projects/programs/activities	60.00%	85.00%	65.00%	35.00%
Conservation prioritized in land use development	55.00%	25.00%	55.00%	25.00%
Regulate wetland disturbance and impact activities	55.00%	45.00%	45.00%	35.00%
Policies on rural character maintaining	55.00%	45.00%	75.00%	40.00%
Permitted use protecting natural system	55.00%	50.00%	40.00%	50.00%
Coordination with local/state/federal agencies	55.00%	5.00%	40.00%	30.00%
Natural or environmental resources described (open space, green space, river corridors)	50.00%	45.00%	45.00%	30.00%
Conservation areas mapped/described	50.00%	45.00%	60.00%	55.00%
Regulations on ecosystem and biodiversity conservation (Wildlife, bird, crane)	50.00%	45.00%	50.00%	45.00%
Natural educational opportunities	50.00%	30.00%	50.00%	50.00%
Implementation timelines/trend	50.00%	40.00%	55.00%	0.00%
Environmental sensitive area described	45.00%	35.00%	50.00%	30.00%
Water resources related description (e.g., quantity and quality, pollution)	45.00%	20.00%	50.00%	25.00%
Water resource protection	45.00%	55.00%	60.00%	35.00%
Conservation projects/programs	40.00%	40.00%	40.00%	60.00%
Conservation design standards and techniques	40.00%	40.00%	55.00%	55.00%
Wetland/water management and restoration	40.00%	30.00%	55.00%	25.00%
Goals for ecosystem/biodiversity protection (Wildlife, bird, crane)	35.00%	10.00%	35.00%	50.00%
Low density design standard and building codes for conservation	35.00%	40.00%	25.00%	15.00%
Stormwater management	35.00%	65.00%	65.00%	20.00%
Maintenance and updating procedure and development continuum	35.00%	70.00%	50.00%	25.00%
Road and transportation standard adjustment for conservation	30.00%	30.00%	35.00%	80.00%

**Table 5:** Mean county-level agreement and 95% county-bootstrap confidence intervals under ternary scoring criteria

Model	Prompt condition	Mean ternary agreement	95% CI
GPT-4o	basic	47.69%	44.43%–51.35%
GPT-4o	optimized	62.20%	58.98%–65.42%
GPT-4o mini	basic	43.35%	38.87%–47.90%
GPT-4o mini	optimized	58.75%	53.66%–63.57%

Phase 1 refined task descriptions and operational instructions in the prompts (Figure 1) and reached an optimization plateau after approximately 30 iterations. On a training-set subset comprising 10 indicators sampled from five categories of the 56-indicator protocol, GPT-4o mini exact-match accuracy increased from 42% to 56%. This initial phase improved the model’s ability to interpret complex plan-evaluation tasks.

**Figure 1:**
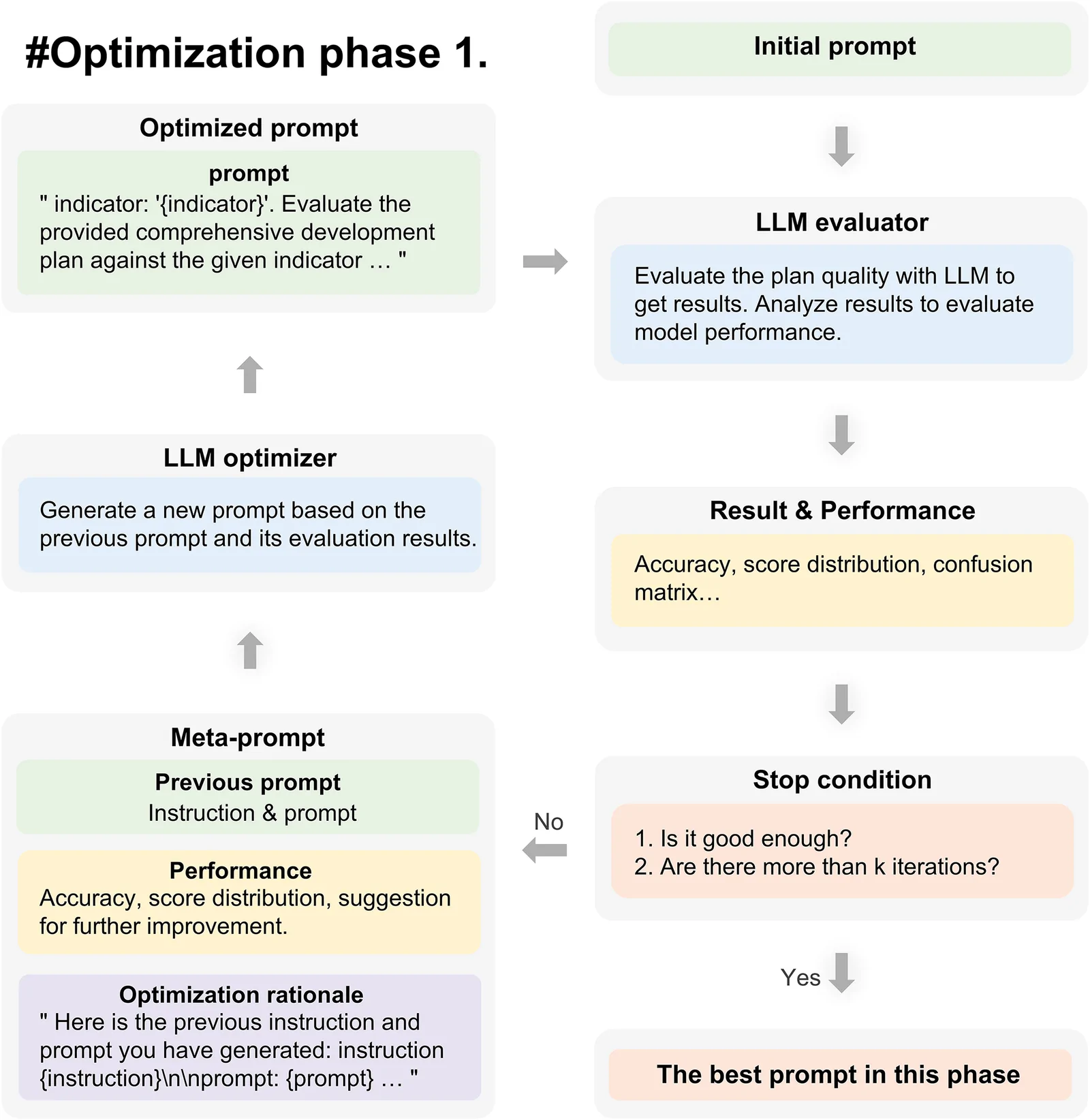
General optimization workflow of phase one

Phase 2 incorporated indicator-specific annotations and detailed scoring criteria (Figure 2) and reached an optimization plateau after approximately 900 iterations. Building on the Phase 1 prompts, manually developed scoring criteria, and knowledge-base-generated annotations, this phase increased mean GPT-4o mini exact-match accuracy from 46.78% to 65.35% across the full training set. The annotations were intended to reduce domain-knowledge barriers, and the refined metrics were intended to operationalize evaluation requirements and broaden the basis for judgment.

**Figure 2:**
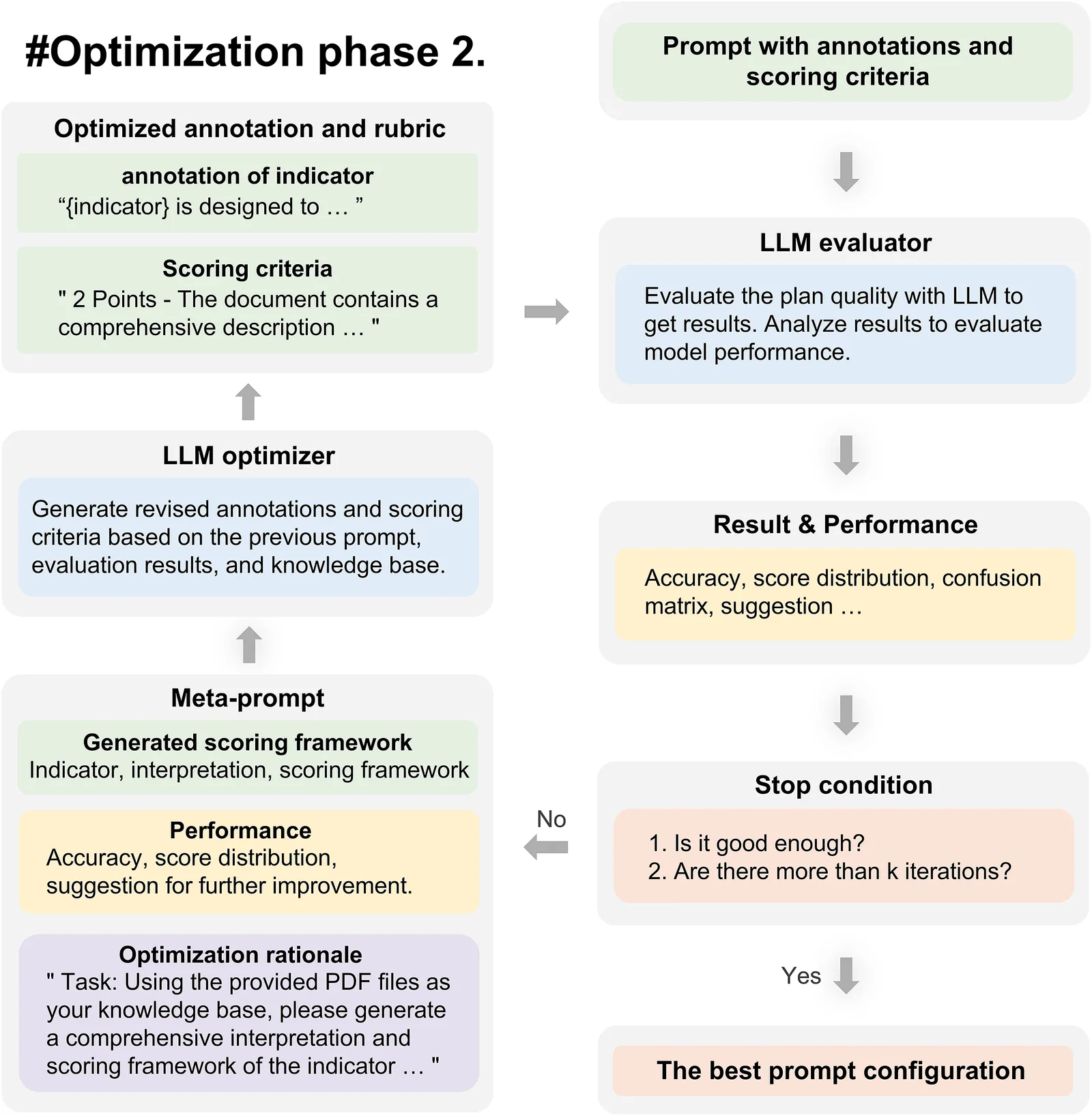
General optimization workflow of phase two

Although optimized annotations and scoring criteria improved exact-match accuracy, the unoptimized combination of annotations and human-written rubrics reduced performance. On the Phase 1 subset, this combination achieved 46% exact-match accuracy, compared with 58% for the Phase 1 optimized prompts and 78% for the Phase 2 optimized framework. These limited development-stage comparisons indicate that the combined optimized framework outperformed the tested unoptimized combinations. The token cost of both optimization phases was approximately $150.

### Discrepancies between GPT and human judgments

Through in-depth analysis of discrepancies between GPT and human judgments, we identified that the scoring errors of ChatGPT predominantly cluster at the demarcation boundaries between 0–1 and 1–2 ratings.

Distinguishing scores of 0 and 1 is particularly consequential because it requires the model to determine whether the content specified by an indicator is addressed in a planning document. Review of these misclassifications indicated that misunderstanding and overgeneralization of indicator requirements accounted for nearly 60% of reviewed error cases. This problem was most apparent for indicators containing domain-specific planning terminology. The model also had difficulty identifying indirect evidence that required professional knowledge or practical experience and recognizing implicit relationships among informational elements. Representative error cases are presented in Table 6. Exploratory review further suggested that some disagreements may reflect uncertainty or oversight in the human reference labels.

**Table 6:** Representative error instances of points 0 and 1

Indicator	County	ChatGPT’s response	Reasons for misjudgment
Low density design standard and building codes for conservation	Butler	### Score: 1 ### Reason: 1. ∗∗General Mention of Environmental Sensitivity:∗∗ The Butler County Comprehensive Development Plan discusses the importance of recognizing environmental sensitivities, such as flood-prone areas, when planning future developments. It suggests restricting certain developments in these regions to mitigate environmental impacts Butler.pdf. 2. ∗∗Basic Reference to Land Use Planning:∗∗ There is a reference to adopting land use plans and zoning practices that integrate sensitivity to environmental capacities, implying a consideration for protecting sensitive areas through planning measures Butler.pdf. 3. ∗∗Lack of Specific Low-Density Standards or Building Codes:∗∗ The plan does not specify low-density design standards or detailed building codes specifically aimed at wetland preservation or clearly linked conservation strategies Butler.pdf. The plan acknowledges the importance of considering environmental capacities in planning and zoning but lacks comprehensive details specifically on low-density design standards for conservation, resulting in a score of 1.	**Manual Score: 0** The Butler County plan mentions development restrictions in environmentally sensitive areas but does not explicitly define low-density standards. GPT may have mistaken general environmental policies for compliance with the indicators but the manual review found a lack of specific connections between low-density requirements and wetlands. Here, GPT overgeneralized by equating environmental sensitivity with low-density standards.
Development rights related strategies	Wayne	### Score: 1 ### Reason: 1. ∗∗Acknowledgment of Low Impact Development (LID)∗∗: The Wayne County Comprehensive Development Plan includes support for Low Impact Development programs, suggesting a general commitment to sustainable development. It specifies LID design options in engineering services contracts and promotes water conservation and urban forestry within subdivision agreements Wayne.pdf. 2. ∗∗Limited Mention of Mixed-Use Development∗∗: While the plan supports some concepts of mixed-use development, such as encouraging redevelopment and infill construction standards, these aspects are not explicitly tied to wetland ecological benefits or conservation within the context of the document Wayne.pdf. 3. ∗∗Need for Direct Wetland Conservation Link∗∗: The LID practices noted, although beneficial for sustainability, are not integrated with explicit objectives for wetland protection. This indicates a need for more focused measures to align such development strategies with wetland conservation efforts Wayne.pdf. In summary, the plan references LID but lacks detailed, explicit integration of mixed-use development strategies directly linked to wetland conservation, resulting in a score of 1.	**Manual Score: 0** **Terminology Misinterpretation:** ChatGPT interpreted “Low Impact Development (LID)” as an ecological conservation strategy but the text did not explicitly establish a connection between LID and wetland protection. **Mixed-Use Development Misunderstanding:** The plan mentions ‘mixed-use development’ but does not specify its positive impact on ecosystems; ChatGPT incorrectly associated it with unrelated policies.
Coordination with NGOs or environmental organizations/research institutes/participants	Hitchcock	### Score: 0 ### Reason: 1. ∗∗Absence of Collaboration with NGOs or Environmental Organizations∗∗: - The Hitchcock County Comprehensive Development Plan does not explicitly reference or detail any collaboration with NGOs, environmental organizations, research institutes, or community participants for the purpose of wetland or broader environmental conservation Hitchcock.pdf. 2. ∗∗Focus on General Conservation Goals∗∗: - While the plan discusses goals and strategies for conservation, these are primarily internal to local government policies and do not involve external partnerships or collaborative efforts with non-governmental entities Hitchcock.pdf. 3. ∗∗Lack of Specific Initiatives or Partnerships∗∗: - The absence of specific initiatives or partnerships with external bodies suggests a missed opportunity to leverage additional resources, expertise, and innovation in addressing conservation challenges Hitchcock.pdf. Due to the lack of explicit references to coordination with NGOs or research institutions, the plan scores 0 for this indicator.	**Manual Score: 1** While the plan contains relevant content, it is not directly linked to wetlands, nor even closely connected to water resource protection. Its primary relevance lies in land use.

Bold text highlights the human reference scores and the subheadings summarizing the reasons for model misjudgment.

The distinction between scores of 1 and 2 depends on the emphasis given to an indicator in the planning text, the specificity of the associated description, and the completeness and feasibility of proposed measures. Determining whether indicator-related content is mentioned is generally less difficult than distinguishing partial from full fulfillment. Review of these errors showed that GPT could conflate superficial mentions with substantive elaboration, assigning high scores to generic descriptions that lacked operational detail. For example, the model could equate statements about regularly reviewing and updating a plan with formal maintenance procedures or treat broad proposals to establish protected areas or regulate livestock farming as fully developed policies despite the absence of implementation frameworks. Class-specific F1 scores support this interpretation (Table 7). Under optimized prompts, ternary F1 scores for GPT-4o were 0.507 for score 1 and 0.497 for score 2; the corresponding values for GPT-4o mini were 0.488 and 0.454. After scores were collapsed into a binary presence/absence outcome, positive-class F1 was 0.700 for GPT-4o and 0.694 for GPT-4o mini. These results are consistent with stronger performance in identifying whether an indicator was addressed than in differentiating partial from full fulfillment.

**Table 7:** Class-specific F1 scores for optimized prompts under ternary and post-hoc binary scoring criteria

Configuration	Ternary F1: Score 0	Ternary F1: Score 1	Ternary F1: Score 2	Binary F1: Score 0	Binary F1: Score 1
GPT-4o/optimized	0.729	0.507	0.497	0.729	0.700
GPT-4o mini/optimized	0.695	0.488	0.454	0.695	0.694

### Difference between 0–1 and 0–1–2 scoring system

Because the distinction between scores of 1 and 2 depends on the degree of compliance, descriptive depth, and completeness of indicator-relevant content, we also examined a coarser binary outcome. In this post-hoc transformation, score 0 denoted the absence of indicator-related considerations, whereas original scores of 1 and 2 were combined into a presence category. Binary item-level agreement was 71.49% for GPT-4o/optimized and 69.46% for GPT-4o mini/optimized. For GPT-4o/optimized, 16 indicators exceeded 80% agreement, and 24 exceeded 70%; the corresponding counts for GPT-4o mini/optimized were 17 and 25. Detailed indicator-level binary agreement results are presented in Table 8. Together with the class-specific F1 results in Table 7, these findings are consistent with stronger performance in presence/absence identification than in the original ternary distinction between partial and full fulfillment.

**Table 8:** Recalculated agreement under binary scoring criteria (0–1) for 56 indicators

Indicator	GPT-4o/optimized	GPT-4o/basic	GPT-4o mini/optimized	GPT-4o mini/basic
Natural or environmental resources described (open space, green space, river corridors)	95.00%	100.00%	100.00%	100.00%
Conservation areas mapped/described	65.00%	73.68%	90.00%	75.00%
Environmental sensitive area described	65.00%	63.16%	75.00%	60.00%
Wetland current status and description (historical loss/gain/degradation/challenges)	70.00%	75.00%	45.00%	50.00%
Wetland definition	95.00%	100.00%	100.00%	85.00%
Wetland maps and classifications	95.00%	85.00%	85.00%	75.00%
Local wetland types/classification	95.00%	90.00%	90.00%	90.00%
Conservation projects/programs	45.00%	75.00%	45.00%	75.00%
Conservation prioritized in land use development	60.00%	55.00%	65.00%	55.00%
Conservation lands identified on land use maps/plans	65.00%	50.00%	55.00%	30.00%
Watershed management programs	75.00%	65.00%	70.00%	40.00%
Water resources related description (e.g., quantity and quality, pollution)	80.00%	78.95%	80.00%	85.00%
Watershed development status (ISA, stormwater)	60.00%	70.00%	70.00%	55.00%
Goals for no net loss	95.00%	78.95%	80.00%	40.00%
Goals for anti-degradation	60.00%	78.95%	55.00%	85.00%
Goals for increasing wetland quantity and quality	70.00%	80.00%	85.00%	60.00%
Goals for ecosystem/biodiversity protection (Wildlife, bird, crane)	65.00%	60.00%	60.00%	75.00%
Environmental sustainability/resilience goal	70.00%	70.00%	65.00%	70.00%
Manmade/constructed wetland and wetland restoration	80.00%	90.00%	90.00%	95.00%
Future land use and zoning regulations	95.00%	100.00%	100.00%	100.00%
Zoning policies for specific areas for protection	80.00%	90.00%	90.00%	95.00%
Low density design standard and building codes for conservation	40.00%	40.00%	45.00%	30.00%
Regulate wetland disturbance and impact activities	55.00%	60.00%	50.00%	35.00%
Prohibit development in environmental sensitive areas	90.00%	85.00%	90.00%	90.00%
Subdivision regulations on open space and park land	80.00%	85.00%	80.00%	85.00%
Policies on rural character maintaining	85.00%	100.00%	95.00%	100.00%
Road and transportation standard adjustment for conservation	30.00%	50.00%	40.00%	90.00%
Conservation design standards and techniques	55.00%	70.00%	65.00%	75.00%
Water resource protection	90.00%	95.00%	85.00%	100.00%
Environmental regulations for natural system protection	90.00%	95.00%	85.00%	90.00%
Regulations on ecosystem and biodiversity conservation (Wildlife, bird, crane)	65.00%	70.00%	65.00%	65.00%
Regulations for buffer zone	80.00%	89.47%	85.00%	55.00%
Wetland/water management and restoration	50.00%	50.00%	65.00%	30.00%
Permitted use protecting natural system	60.00%	85.00%	50.00%	80.00%
Stormwater management	45.00%	42.11%	65.00%	30.00%
Water related BMPs	65.00%	60.00%	65.00%	15.00%
Local tax incentives to support conservation	70.00%	70.00%	75.00%	60.00%
Mixed use development/Low impact development	65.00%	40.00%	60.00%	15.00%
Development rights related strategies	70.00%	50.00%	40.00%	15.00%
Clustered development	80.00%	60.00%	75.00%	40.00%
Density bonuses	90.00%	90.00%	70.00%	65.00%
Conservation easement	90.00%	90.00%	80.00%	75.00%
Land acquisition program for conservation	85.00%	75.00%	85.00%	85.00%
Financial/economic incentive for conservation	70.00%	63.16%	65.00%	60.00%
Conservation education/outreach projects/programs/activities	60.00%	45.00%	75.00%	55.00%
Natural educational opportunities	50.00%	70.00%	55.00%	55.00%
Waterfront recreational sites for education/educational facilities	70.00%	50.00%	70.00%	25.00%
Link local approvals with 404 permit and/or 401 certifications/swampbuster/title 117	95.00%	89.47%	85.00%	78.95%
Coordination with local/state/federal agencies	70.00%	95.00%	40.00%	90.00%
Coordination with NGOs or environmental organizations/research institutes/participants	35.00%	45.00%	35.00%	80.00%
Integration with other plans/policies/programs	75.00%	94.74%	50.00%	90.00%
Implementation timelines/trend	50.00%	25.00%	55.00%	25.00%
Wetland-related task force/committee	95.00%	95.00%	95.00%	95.00%
Maintenance and updating procedure and development continuum	70.00%	80.00%	60.00%	78.95%
Plan effectiveness on conservation monitoring	90.00%	63.16%	50.00%	65.00%
Implementation of conservation development techniques/practices	63.16%	25.00%	45.00%	15.00%

ChatGPT performed relatively well on indicators that relied on surface-level information similarity, yet it faced greater difficulty when evaluation required assessment of logical coherence, feasibility, or goal relevance. For the indicator “Coordination with NGOs or environmental organizations/research institutes”, ChatGPT overlooked descriptions of collaboration with entities such as state historic sites and federal agencies in Antelope County, as well as similar references to public facilities, community organizations, and energy offices. These cases suggest that the model relied heavily on explicitly stated keywords and information directly represented in the prompt. Some organizations were classified as non-NGO entities, yielding score-0 evaluations despite evidence of cooperation. These errors reflect limitations in semantic interpretation, although they do not fully explain the most unreliable cases in which model-human agreement approached random levels.

For a small subset of counties, moderate percent agreement coincided with near-random Krippendorff’s alpha values; some alpha values were below zero. Counties with poor alpha performance generally had lower-quality plans. York County, which had the lowest alpha, received a human-assigned score of 0.30, compared with a statewide mean of 0.84, and all component scores fell below the state mean. Many indicators in the York County plan challenged both the two initial human coders and the secondary reviewer. Post-hoc review suggests three contributing factors: (1) lower professional rigor than in more mature planning documents; (2) a 10-year planning horizon that differed from the more common 20-year horizon and was associated with substantive policy differences; and (3) a nonstandard structure, including the absence of a table of contents and an implementation chapter, which increased variation in how coders and the model located and interpreted relevant information.

Nebraska is a relatively small state, and Planning Commissions in many sparsely populated counties are composed largely of citizen volunteers. Their professional capacity may therefore differ from that of planning agencies in major metropolitan areas. In contrast, the plans examined by Fu et al. were drawn from large metropolitan or micropolitan counties, where planning agencies generally had greater professional resources and produced higher-quality documents. Despite these contextual differences, our findings align with and extend those of Fu et al.: counties with stronger planning capacity, larger populations, and more robust economies, such as Lancaster County, showed higher model-human agreement. In these contexts, machine evaluation may provide useful support for human coding. When underlying plan quality was low, however, model performance did not overcome the resulting limits on evaluation reliability.

Furthermore, the retrieval mechanism exhibits limited generalization capability for processing implicitly associated information, leading to scoring inconsistencies where discrepancies arise not from ChatGPT’s judgment errors but from the system’s failure to retrieve content considered critical in human evaluations. Although these represent isolated cases, we emphasize that such limitations warrant particular attention when implementing language models in practical plan evaluation scenarios.

Language models can serve as useful evaluation assistants when human scoring is inconsistent. Comparison of the reference texts and evaluation criteria used in human and ChatGPT assessments suggests two mechanisms. First, human evaluators may apply standards inconsistently when indicator descriptions are ambiguous. Model-generated scores and rationales can be compared with the predefined requirements to support human review. Second, human evaluators may overlook indicator-relevant content, particularly when it depends on indirect logical connections. Retrieval-based systems can help identify such content. By comparing ChatGPT’s scoring rationales with the corresponding text blocks, human evaluators can assess whether a model decision is supported by retrieved evidence and identify potential omissions or inaccuracies in the reference labels. In this study, the analysis of final model-human disagreements identified relevant planning content that had remained overlooked despite the earlier human review. These findings informed the interpretation of discrepancies and the discussion of residual uncertainty in the human reference labels. They did not lead to changes in the reference scores used for the reported analyses or to further prompt optimization.

During Phase 2 optimization, a small number of interim results showed lower agreement under optimized prompts. Review indicated that some added annotations and independent scoring criteria diverged directionally or quantitatively from the intended evaluation objectives. For example, prompts requiring an “explicit mention of wetland protection” could trigger overly strict binary assessments when contextually relevant implicit content, accepted by human evaluators, was present. In such cases, ChatGPT systematically classified the indicator as noncompliant. These suboptimal interim specifications were generally addressed through subsequent automated adjustments by the optimizer.

In final prompt configurations, three indicators evaluated by the ChatGPT-4o mini model still exhibited significant performance reduction, whereas the ChatGPT-4o model demonstrated substantially mitigated issues in the corresponding evaluations of these indicators. These differences may reflect differences in model capabilities, although the present study did not isolate the contributions of model characteristics and retrieved evidence. After optimization, all prompts, indicator annotations, and scoring criteria underwent manual verification for alignment with the evaluation requirements. This verification concerned the prompt materials, and the human reference scores remained unchanged. However, nuanced semantic distinctions in prompt phrasing present inherent limitations to human auditing processes.

## Discussion

### Effectiveness of LLMs to evaluate plans

Current ChatGPT models, including the lower-cost GPT-4o mini and the higher-performing GPT-4o, remain limited in conducting planning-text evaluation under ternary scoring criteria. Under the less ambiguous binary criteria commonly used in practice, they can support assessment of selected indicators. Based on these findings, we offer the following recommendations for researchers using ChatGPT in plan evaluation.

First, language models are highly sensitive to prompt formulation. Rule-of-thumb prompt design can diverge from the intended evaluation objective because small semantic variations may affect model behavior. Systematic iterative testing and targeted prompt optimization are therefore important for developing prompts that operationalize evaluation criteria precisely. We recommend using a structured iterative optimization framework tailored to the specific evaluation task.

Second, ChatGPT tends to rigidly adhere to the literal requirements of prompts for “strict” evaluation while simultaneously misinterpreting and overgeneralizing specialized terminology. This results in ChatGPT demonstrating strong performance when assessing well-differentiated indicators yet underperforming on indicators requiring professional expertise to capture connections between indirect evidence and content. We recommend conducting pilot testing on evaluation indicators or categorizing them based on empirical evidence before deploying ChatGPT or other language models in evaluation tasks. Indicators with higher professional demands should be identified through specialization requirements analysis. Moreover, when processing such specialized indicators with language model assistance, additional human scrutiny must be implemented to ensure validity.

Given the current performance of language models in plan evaluation, we recommend their use as auxiliary tools within established research workflows. Retrieval-based matching can identify text segments relevant to indicators, annotations, and scoring rubrics, thereby reducing human oversight of important planning information. Model-generated scores and rationales may provide an additional reference for indicators that human evaluators find ambiguous or difficult to score. These functions support the use of language models as scoring references or secondary-review tools.

Finally, clarifying how ChatGPT discerns the ambiguous boundaries between different scoring levels for each indicator remains one of the most critical directions for enhancing its applicability in evaluation frameworks. Current limitations in ChatGPT’s intermediate scoring judgments primarily involve misinterpretation and overgeneralization of indicators, along with erroneously categorizing superficial mentions as substantive discussions. Potential solutions include (1) providing few-shot exemplars, both validated cases and counterexamples, or sample texts containing potential indirect associations and (2) simplifying evaluation complexity by decomposing nonbinary quantitative indicators into sequential evaluation phases.

Based on the observed model-human agreement and the professional expertise required for each indicator, we classified the indicators used in this study into three applicability categories. The classification is presented in Table 9.

a) Category I (Reliable): These indicators showed higher agreement under the study-specific classification rule but model-generated scores still require human verification.
b) Category II (Verification Required) comprises indicators with moderate agreement that remains below the threshold for reliable automated assessment. Language-model outputs for these indicators require rigorous validity checks.
c) Category III (Unusable) comprises indicators with critically low model-human agreement. For these indicators, language models should be used only to assist human review after initial human coding.

**Table 9:** Reproducible applicability classification for coding protocol in this study

Classification	Indicator
Category I: Reliable	Wetland definition
	Wetland-related task force/committee
	Local wetland types/classification
	Wetland maps and classifications
	Goals for no net loss
	Future land use and zoning regulations
	Link local approvals with 404 permit and/or 401 certifications/swampbuster/title 117
	Conservation easement
	Zoning policies for specific areas for protection
	Density bonuses
	Land acquisition program for conservation
	Manmade/constructed wetland and wetland restoration
	Prohibit development in environmental sensitive areas
	Goals for increasing wetland quantity and quality
	Watershed management programs
	Regulations for buffer zone
	Waterfront recreational sites for education/educational facilities
Category II: Verification Required	Environmental regulations for natural system protection
	Local tax incentives to support conservation
	Policies on rural character maintaining
	Clustered development
	Financial/economic incentive for conservation
	Plan effectiveness on conservation monitoring
	Conservation education/outreach projects/programs/activities
	Watershed development status (ISA, stormwater)
	Subdivision regulations on open space and park land
	Environmental sustainability/resilience goal
	Water related BMPs
	Mixed use development/Low impact development
Category III: Unusable	Conservation lands identified on land use maps/plans
	Conservation areas mapped/described
	Conservation prioritized in land use development
	Development rights related strategies
	Water resource protection
	Implementation timelines/trend
	Implementation of conservation development techniques/practices
	Regulations on ecosystem and biodiversity conservation (Wildlife, bird, crane)
	Stormwater management
	Natural educational opportunities
	Regulate wetland disturbance and impact activities
	Natural or environmental resources described (open space, green space, river corridors)
	Water resources related description (e.g., quantity and quality, pollution)
	Environmental sensitive area described
	Integration with other plans/policies/programs
	Conservation design standards and techniques
	Wetland current status and description (historical loss/gain/degradation/challenges)
	Wetland/water management and restoration
	Coordination with local/state/federal agencies
	Permitted use protecting natural system
	Maintenance and updating procedure and development continuum
	Goals for anti-degradation
	Conservation projects/programs
	Goals for ecosystem/biodiversity protection (Wildlife, bird, crane)
	Road and transportation standard adjustment for conservation
	Low density design standard and building codes for conservation
	Coordination with NGOs or environmental organizations/research institutes/participants

Note: Mean agreement is averaged across GPT-4o/optimized and GPT-4o mini/optimized. The joint agreement score is the lower of the mean ternary and mean binary agreements. Classification thresholds are ≥70%, 60%–<70%, and <60%.

### API token costs of prompt optimization

Prompt optimization with GPT-4o mini was associated with improved agreement in this study. The two-phase GPT-4o mini optimization workflow involved approximately 1,000 iterations and incurred approximately $150 in token expenditure. Both GPT-4o and GPT-4o mini showed improved agreement under optimized prompts. The OpenAI API-based framework can be adapted to other evaluation tasks. In practice, researchers may first conduct manual evaluations on a random subset of targets and then use the resulting labels and task-specific coding protocol to refine prompts iteratively.

### Beyond plan evaluation

Beyond the specific domain of plan quality evaluation, the present findings contribute to a nascent yet expanding body of research employing LLMs for policy document analysis and social science text evaluation. Prior investigations have examined LLM applications in climate policy coherence assessment, legislative bill analysis, and regulatory impact evaluation. A recurring observation across this literature is that the principal impediment to reliable automated evaluation resides not in the generative fluency of the models but rather in the misalignment between their generic pretraining objectives and the domain-specific evaluative constructs required for specialized tasks. The difficulties identified in the present study, wherein LLMs exhibited limited capacity to interpret specialized planning terminology and to integrate implicit evidence distributed across chapters, are consistent with those documented in adjacent policy evaluation domains. The prompt optimization framework and iterative refinement methodology advanced herein may thus offer transferable strategies for scholars engaging with human-coded rubrics and extensive, unstructured government documents across diverse policy contexts. Furthermore, the finding that systematic prompt engineering, even when modest in scope, can yield non-trivial improvements in model-human agreement provides an empirically grounded reference for social science researchers who wish to incorporate LLMs into their content analysis workflows.

This study developed a prompt-optimization methodology for plan evaluation. Using language models as optimizers, the approach iteratively refined prompts to improve GPT-4o performance in plan evaluation. We used comprehensive plans from 25 Nebraska counties (five for prompt development and twenty for validation) and compared manual and ChatGPT evaluations under basic and optimized prompt conditions on the validation set. Validation outputs showed improved model-human agreement under optimized prompts. Under ternary scoring, GPT-4o achieved agreement of at least 80% for only a subset of indicators and could not replace human coders. Under the post-hoc binary benchmark, 24 of 56 indicators exceeded 70% agreement for GPT-4o/optimized and 25 exceeded 70% for GPT-4o mini/optimized; 16 and 17 indicators, respectively, exceeded 80%. Neither GPT-4o nor GPT-4o mini can independently replace human evaluators for ternary scoring, although binary criteria yielded stronger agreement for a subset of indicators. Under optimized prompts, GPT achieved relatively high agreement with human reference scores on a subset of indicators, although agreement varied across indicators and scoring schemes. Human cross-validation remains essential for assessing the accuracy of model outputs. Prompt quality substantially influences evaluation results; tailored prompt optimization is therefore important for plan-quality evaluation.

### Limitations of the study

This study used comprehensive plans from 25 Nebraska counties, with five plans used for prompt development and 20 for held-out validation (Table 10). The development process used a 10-indicator subset in Phase 1 and indicator-specific optimization across the 56-indicator protocol in Phase 2. Repeated optimization on a small development set may overfit prompt wording to the documents and label distributions represented in that set. In addition, the development and validation plans came from the same state, and the study did not include external validation using plans from other geographical or institutional settings. The observed performance therefore does not establish generalizability beyond the study setting. Future studies should use larger and more diverse development sets and independently evaluate the resulting prompts across regions, planning systems, and document types.

**Table 10:** Development-validation plan split and available document characteristics

Split	Unit	Jurisdiction in document	Year or horizon	Pages	Source format
Development	Cherry	County	2000	97	PDF
Development	Douglas	County	2006	122	PDF
Development	Keith	County	2003	110	PDF
Development	Saunders	County	2004	112	PDF
Development	Sheridan	County	2014	274	PDF
Validation	Antelope	County	2012	63	PDF
Validation	Buffalo	County	2022	85	PDF
Validation	Butler	County	2007–2020	78	PDF
Validation	Dakota	County	2014	91	PDF
Validation	Dawes	County	Not reliably recovered	96	PDF
Validation	Furnas	County	2016	116	PDF
Validation	Greeley	County	2015	83	PDF
Validation	Hitchcock	County	2001	158	PDF
Validation	Howard	County and communities	2015	141	PDF
Validation	Kearney	County and communities	2010	83	PDF
Validation	Kimball	City	2024	123	PDF
Validation	Knox	County	Not reliably recovered	105	PDF
Validation	Lincoln	County	2012–2030	190	PDF
Validation	Nance	County	2022	109	PDF
Validation	Perkins	County	2013	104	PDF
Validation	Red Willow	County label in source filename	Not reliably recovered	77	PDF
Validation	Seward	County	2007–2030	175	PDF
Validation	Valley	County	2014	53	PDF
Validation	Wayne	City	2017–2027	156	PDF
Validation	York	County	2013	74	PDF

Each of the four model-prompt configurations underwent one full evaluation on the held-out validation set. Consequently, the study does not quantify variability across repeated full validation runs. Development-stage checks on the five development plans cannot establish test-retest reliability on the held-out validation set. Future studies should document model-version identifiers and execution settings, conduct repeated validation runs under fixed conditions, and report score-level test-retest agreement and associated uncertainty.

Information availability differed between the automated workflow and human coding. Human coders could inspect the original planning documents, including maps and other graphical materials, whereas the automated workflow was restricted to parseable text. Performance on indicators that depend on visual evidence should therefore be interpreted under this text-only constraint and does not establish multimodal planning-assessment capability. Future studies should provide comparable text-and-image evidence to human coders and models and evaluate performance on visually dependent indicators separately.

The three applicability categories summarize patterns observed in this study. Whether the same grouping holds for other plans or model configurations requires further evaluation.

The two prompt-optimization phases incurred approximately US$150 in API token costs. Applying this workflow also requires time for preparing human reference labels, developing prompts, and checking model outputs. These inputs were not tracked systematically in this study and should be considered alongside API expenditure when assessing the resources needed for practical use.

Currently, language models are emerging continuously. Our research specifically focuses on ChatGPT-4o and its variant, ChatGPT-4o mini. Future investigations should prioritize designing cross-model comparative experiments to evaluate the performance of various language models in planning evaluation domains, aiming to identify the balance between capability and computational expenditure of optimal models. Additionally, developing domain-specialized models through fine-tuning different language architectures for plan evaluation applications remains an underexplored research frontier. Such fine-tuning on curated corpora of planning documents could enable open-source LLMs to internalize domain-specific terminology and evaluative heuristics, potentially achieving higher accuracy and lower inference costs compared to prompt-based approaches. Furthermore, more sophisticated RAG architectures, including iterative retrieval with self-verification mechanisms, hierarchical indexing tailored to multi-section comprehensive plans, or knowledge graph-enhanced retrieval, may better capture the implicit logical connections and cross-references that are characteristic of these documents and often elude standard single-pass retrieval.

Language models can serve as assistants within multiphase planning evaluation workflows. Beyond the evaluation stage, using language models as reference tools during review may help reviewers identify cases that need further attention. After manually resolving discrepancies between GPT-generated and human evaluations, we conducted secondary reviews using GPT to detect potential human errors in discordant cases. These reviews suggested that language models could help identify some potential human errors, although they also flagged some correct human evaluations as errors. Language models may therefore support secondary review, with suggested corrections checked against the source documents.

Generative language models remain prone to errors, and their outputs require critical human scrutiny. Higher agreement under the post-hoc binary transformation reflects performance on a coarser scoring task and does not establish reliable autonomous plan evaluation. The findings support further investigation of model-assisted evidence retrieval, preliminary screening, and secondary review with human verification. The reliability of these uses should be evaluated directly before deployment.

## Resource availability

### Lead contact

Further information and requests for resources should be directed to and will be fulfilled by the lead contact, Ligang Zhang (zlg@hznu.edu.cn).

### Materials availability

No new unique materials were generated in this study.

### Data and code availability

Data reported in this study have been deposited in Mendeley Data and are publicly available at Mendeley Data: https://doi.org/10.17632/y2pgcb4t5r.1 (version 1).

Custom code, prompt templates, and the instruction framework used in this study are available at https://github.com/HaishanLinHZNU/LLM_Plan_Evaluation.

Any additional information required to reanalyze the data reported in this paper is available from the lead contact upon request.

The shared resolved files contain successfully parsed records from 40 archived plan units; the analyses reported here use only the 20 held-out validation plans. Retrieved text chunks and API message and run-step logs are excluded from the shared files. The code archive and resolved outputs are identified in the key resources table.

## Acknowledgments

This research was supported by the Hangzhou Joint Fund of Zhejiang Provincial Natural Science Foundation of China under grant no. LHZQN25D010001. Each of the authors’ respective institutions contributed, however, by allowing time for the researcher’s work on data collection, calculations, analyses, and writing. The work is, nevertheless, based on data material developed in previous projects where funding was received from U.S. Environmental Protection Agency, United States, grant no. CD 97790401 and CD 97763501. The authors would also like to thank Dr. Zhenghong Tang for his valuable contributions to wetland conservation in Nebraska, U.S.A.

## Author contributions

Conceptualization, L.Z. and H.L.; methodology, L.Z., and H.L; investigation, H.L., L.Z., and Y.Z.; data curation, H.L.; writing – original draft, H.L.; writing – review and editing, H.L., B.Z., X.L., Y.Z., X.T., and L.Z.; funding acquisition, L.Z.; resources, H.L., and B.Z.; supervision, L.Z.

## Declaration of interests

The authors declare no competing interests.

## Declaration of generative AI and AI-assisted technologies in the manuscript preparation process

During the preparation of this work, the authors used ChatGPT (OpenAI) for language editing and improving the clarity of the manuscript. The authors reviewed and edited the output as needed and take full responsibility for the content of the published article.

## STAR★Methods

### Key resources table

**Table:** 

REAGENT or RESOURCE	SOURCE	IDENTIFIER
**Deposited data**		

Dataset for wetland conservation evaluation in local plans	Zhang, Ligang (2026), Mendeley Data, version 1	Mendeley Data: https://doi.org/10.17632/y2pgcb4t5r.1
Plans of 20 counties in Nebraska, USA	Zhang et al.	https://doi.org/10.1080/20964129.2022.2070550
Result of Evaluation by human researchers	Zhang et al.	https://doi.org/10.1080/20964129.2022.2070550
Coding protocol	Zhang et al.	https://doi.org/10.1080/20964129.2022.2070550
Custom code, prompt templates, and instruction framework	This paper	https://github.com/HaishanLinHZNU/LLM_Plan_Evaluation Accompanying archive: LLM_Plan_Evaluation-main_revised.zip Configuration file: evaluation_configurations.json
Archived model evaluation outputs	This paper	Four resolved JSON files in the accompanying revision package; filenames listed in evaluation_configurations.json

**Software and algorithms**		

OPRO	Yang et al.	https://api.semanticscholar.org/CorpusID:261582296
Python 3.11	Python Software Foundation	https://www.python.org/; RRID: SCR_008394
Excel 2016	Microsoft Corporation	https://www.microsoft.com; RRID: SCR_016137

### Method details

We designed a series of experiments to answer the three research questions. First, we used ChatGPT to evaluate sample plans using a three-point scale, testing its assessment capabilities against standardized criteria. We then developed a prompt framework through prompt engineering and conducted two rounds of iterative optimization using the language model as both optimizer and evaluator. This process enables the model to progressively refine its understanding of evaluation details, ultimately enhancing its effectiveness in planning evaluation tasks to overcome two major barriers in evaluation practices.

Prompt development was conducted exclusively with GPT-4o mini, which served as both the prompt optimizer and the development-stage evaluator; GPT-4o was not used during prompt development. Final validation comprised four configurations: GPT-4o/basic, GPT-4o/optimized, GPT-4o mini/basic, and GPT-4o mini/optimized. Each configuration evaluated 20 validation plans across 56 indicators, yielding 1,120 planned plan-indicator evaluations per configuration. The archived result filenames record run starts for GPT-4o/optimized, GPT-4o/basic, GPT-4o mini/basic, and GPT-4o mini/optimized on February 23, February 25, February 27, and March 1, 2025, respectively. Table 1 reports the four configurations, their shared retrieval and generation settings, and these recorded run-start dates.

#### Research data

This study builds on our previous plan-quality evaluation research and focuses on county, city, and natural-resources-district comprehensive plans in Nebraska, USA. We applied an enhanced component-indicator scoring system, derived from prior work, to assess the integration of wetland conservation into local plans. The system comprises five components and 56 indicators. County-level comprehensive plans from the previous study were independently evaluated by two researchers, yielding Krippendorff’s alpha above 0.80 and indicating strong inter-coder reliability.^40^ To align the dataset with the present research objectives, we evaluated only county-level comprehensive plans. Table 2 lists all components and indicators.

The county-level comprehensive plans collected from Nebraska ranged from 51 to 292 pages. Text processing divided each document into overlapping chunks of up to 800 tokens, with a 400-token overlap between adjacent chunks. Both prompt optimization and final evaluation used a retrieval cap of 20 semantically relevant text chunks per plan–indicator query (file_search.max_num_results = 20). Evaluating all indicators in the coding protocol for a single plan therefore required substantial processing. Compared with the approach of Fu et al.,^8^ which processed every small text chunk for every indicator, our approach reduced computational overhead. The dataset comprised 25 plans drawn from 40 available Nebraska county-plan records: five development plans and 20 validation plans. Table 10 reports the realized development–validation split and available document characteristics.

Before prompt optimization began, a secondary expert audit was conducted on all plans assigned to the development and validation sets, following the same coding protocol as the initial human evaluation. This independent re-evaluation followed the same coding protocol and achieved Krippendorff’s alpha (α) indicative of strong inter-coder agreement, comparable to that obtained in the initial round. The audit identified and corrected a number of scoring oversights and inconsistencies that were present in the first-round evaluations. Following completion of this audit, the resulting human reference scores were fixed and used throughout prompt optimization and final validation. Nevertheless, it is important to acknowledge that even after three independent coders, two from the initial study and one secondary reviewer, had examined the materials, some residual human error and ambiguity inevitably persisted. This intractability stems from the inherent complexity and subjectivity of plan quality evaluation, particularly when applied to documents of varying professional rigor. Indeed, the difficulty of achieving perfect human consensus on such tasks constitutes one of the primary motivations for exploring whether optimized LLM-based evaluation frameworks can offer a more consistent and scalable alternative.

Additionally, we collected supporting documents, including standard references used during the coding protocol development and wetland-related guidelines. These documents serve as a knowledge base to enhance model performance and ensure the evaluation results align with those of human coders. They provide reference materials and factual foundations for optimizing the prompt design.

#### Retrieval mechanism & hyperparameters

The retrieval mechanism determines which portions of a plan document are available to the language model and is therefore central to evaluation fidelity. Retrieval used the default configuration of OpenAI’s File Search tool wherever possible, with limited parameter changes to support reproducibility. The embedding model was text-embedding-3-large with 256 dimensions. Documents were divided into 800-token chunks with a 400-token overlap, and retrieval combined keyword and semantic vector search. The default ranker (auto, equivalent to default_2024_08_21) reranked retrieved results by relevance. During final evaluation, all four configurations used the OpenAI Assistants API File Search tool with the same explicit retrieval cap (file_search.max_num_results = 20). The underlying similarity metric is not exposed by the API; the ranker integrates vector similarity and keyword matching to determine relevance.

For all four final-evaluation configurations, the reported generation setting was temperature = 0.0. Temperature was selected to reduce sampling variability, although it does not guarantee fully deterministic or perfectly repeatable outputs. The updated evaluation scripts explicitly set temperature = 0.0 and file_search.max_num_results = 20. Other generation parameters were not explicitly overridden in these scripts. Complete run-level sampling metadata were not retained in the archived output files; evaluation_configurations.json documents the manuscript-reported settings and the updated runner configuration.

#### LLM evaluating plans

In this study, we used ChatGPT-4o with engineered prompts system to evaluate plans and compared its outputs with results evaluated by a human coder. Through iterative prompt optimization, we enhanced the model performance in planning evaluation tasks.

In the initial phase, we developed code using the OpenAI Assistants API to interact with GPT-4o and GPT-4o mini and preprocessed 25 plan samples. Each document was chunked and vectorized to support retrieval during evaluation, then stored as a vector store on OpenAI’s servers. For each plan evaluation, ChatGPT retrieved the most relevant text chunks in response to the prompt and generated an answer solely from that retrieved context. The workflow indexed and retrieved parseable text only; embedded maps, figures, and image labels were not provided as visual inputs.

Throughout all evaluation steps, this study consistently applied a ternary scale to assess each indicator, aligning with our prior research. During evaluation, human coders code each indicator in the protocol with a 0, 1, or 2, corresponding to noncompliance, partial compliance, or full compliance with that metric, respectively.

Moreover, as a language model, ChatGPT inherently produces textual responses instead of structured numerical outputs, which makes sifting through generated text manually to extract evaluations a necessity. This falls short of our needs. Language models function by learning lexical probability distributions within language.^41^^,^^42^ This enables them to predict the likelihood of word sequences, thereby facilitating the generation of novel text^43^ or the completion of provided text.^44^ The process of transforming input into output constitutes a “black box,” which lacks interpretability.^45^ To address this limitation, we designed a structured output framework for ChatGPT to produce structured responses. It consists of two key components: (1) a numerical score in structured form which can be extracted directly and compared with result evaluated by a human coder and (2) a rationale detailing factual text passages and logical reasoning behind the score.

In its own right, the interpretability of language model outputs serves as a debugging aid applicable for enhancing model performance on various tasks.^46^^,^^47^ We gain deeper insights into scoring decisions by analyzing retreated factual text chunks and evaluation rationale. The entire methodology of this study operates within this structured output paradigm.

To improve language-model understanding of plan evaluation, we used role-based prompt engineering and assigned distinct operational roles to ChatGPT for plan evaluation, prompt optimization, and coding-protocol annotation refinement. Each assistant instruction set specified the model’s operational role, task objectives, procedures, permissible tools, and expected response format. This role-specific configuration supported targeted task execution while maintaining a consistent output format.

Using the framework described above, we first conducted automated plan evaluations under a baseline prompt condition. For each indicator, the user-level request was: “indicator: ‘{indicator}’. Evaluate the provided comprehensive development plan against the given indicator.” This request was paired with a shared assistant instruction containing the common three-point scoring definitions and a fixed output template requiring a score under “### Score” and supporting statements under “### Reason.” We then iteratively optimized the shared instruction and indicator-level prompt materials using training-set performance.

Our automated evaluation procedure comprises the following stages: (a) loading the corresponding vector store for the planning document to the ChatGPT assistant, (b) constructing a prompt for each indicator as input to ChatGPT to evaluate plan, (c) parsing the response to extract evaluation scores, rationales, and referenced text chunks, (d) iteratively executing operations (a)–(c) for all plans and every indicator in the coding protocol, and (e) conducting comparative analysis between ChatGPT and a human coder’s evaluation.

We quantified planning-evaluation performance using exact-match accuracy against human evaluations as the primary development-stage metric. Higher values indicated closer agreement with human coding. The study used GPT-4o to automate plan evaluation through document chunking, vector storage, and structured prompt engineering. The model produced standardized scores with interpretable rationales, which were compared with human evaluations to guide iterative refinement. Task-specific instructions, structured output schemas, and phased prompt optimization supported the accuracy and interpretability of the resulting evaluations.

#### LLM optimization

Prompt development comprised two stages. Phase 1 refined the shared role instruction and common prompt components. Phase 2 used the plan-evaluation knowledge base to generate and evaluate indicator-specific interpretations and three-point scoring frameworks. GPT-4o mini served as both optimizer and development-stage evaluator throughout prompt development; GPT-4o was not used in this process. Candidate performance was assessed only against manually coded labels for the five development plans. The 20 validation plans were held apart from prompt development and were not inspected during optimization. Both phases drew on the approach of Yang et al.,^48^ which uses language models as dual-function optimizers and verifiers, including zero-shot prompting with heuristic phrases such as “let’s think step by step.”

Our method structurally emulates generative adversarial networks by using language models as both optimizer and validator. The validator receives prompts and role instructions, evaluates plans, and produces outputs that are compared with human coding to derive: (1) exact-match accuracy, (2) score-specific systematic bias, including inflation or deflation relative to human benchmarks, and (3) optimization recommendations for prompts and role instructions. A meta-prompt integrates the validator output, the original prompt, and the optimization task description and is then provided to the optimizer to generate a refined prompt. This cycle continues until accuracy reaches a predefined threshold or successive iterations yield no meaningful improvement.

For Phase 2, the meta-prompt incorporated the current retained interpretation and scoring framework, the indicator name, the performance profile of the current framework, and model-generated recommendations. The performance profile included accuracy, mean error, mean squared error (MSE), standard deviation of errors, counts of higher and lower scores, counts of excessive differences, and the confusion matrix. The optimizer generated a candidate interpretation and scoring framework in a prescribed tagged format, after which the development-stage evaluator assessed the candidate on the five development plans.

Each indicator was permitted up to seven candidate generations. A candidate replaced the retained version when it achieved higher accuracy or, when accuracy was equal, lower MSE. Candidates could perform worse than earlier versions and were retained only when they satisfied this comparison rule.

Each iteration of the optimization process required a full evaluation on the training set. During Phase 2, which refined indicator annotations and scoring criteria, each indicator underwent 6–7 optimization iterations.

##### Optimization for prompt and instruction

Language models demonstrate pronounced sensitivity to prompt formulations,^38^ with their performance being contingent upon prompt quality to a certain extent. Consequently, our preliminary optimization prioritized the refinement of both the instructions utilized by the model and the input prompts. The primary objective of this phase is to identify optimal instructions and prompt configurations that maximize model efficacy. The complete optimization workflow of this phase is formally delineated in Figure 1.

The optimization workflow for prompt and instruction is identical to the aforementioned procedure:

a) Prompt Assessment: The candidate prompt is input to the validator to evaluate plans in the training set, generating evaluation results. These results are analyzed to determine the model’s performance when using the prompt.
b) Meta-prompt Construction: The candidate prompt, its performance profile, and the optimization directives are synthesized into a meta-prompt.
c) Prompt Optimization: The meta-prompt is fed into the optimizer to produce an improved candidate prompt.
d) Iterative Optimization: For indicator-specific annotation and scoring-framework optimization, each indicator was permitted up to seven candidate generations. Candidate retention followed a lexicographic rule: higher accuracy was preferred and, when accuracy was tied, lower MSE was preferred. At an optimization plateau, Beichen Zhang (BZ) and Yang Zhang (YZ) assessed whether retained candidates conformed to the human coding standard and contained biased or leading language. A review-approved version could proceed to subsequent use. When a candidate did not pass review, the workflow reverted to the preceding review-approved version. Human review at this stage assessed whether the candidate materials accurately represented the evaluation requirements. The human reference scores remained fixed during this process.

Throughout this optimization phase, all indicators in the coding protocol used the same prompt and instruction during evaluation. The resulting performance metrics therefore represent overall exact-match accuracy and scoring bias across the full training set.

Phase 1 used a 10-indicator subset to develop the shared role instruction and common prompt components. The 56-indicator coding protocol comprised five indicator categories, from each of which two indicators were selected without replacement using Python random.sample. No indicators were selected manually. The realized subset included: (1) natural-resource description: Conservation areas mapped/described and Natural or environmental resources described (open space, green space, river corridors); (2) planning and management: Wetland/water management and restoration and Future land use and zoning regulations; (3) conservation goals: Goals for ecosystem/biodiversity protection (Wildlife, bird, crane) and Environmental sustainability/resilience goal; (4) coordination: Coordination with local/state/federal agencies and Coordination with NGOs or environmental organizations/research institutes/participants; and (5) implementation and follow-up: Maintenance and updating procedure and development continuum and Implementation of conservation development techniques/practices. The sampling script did not set a random seed.

##### Adding annotation and scoring criteria for indicators

While indicators for evaluation may appear superficially concise (often formulated as single statements), they often encompass a multitude of underlying related concepts. Human coders achieve operational proficiency through sustained learning, with comprehensive understanding of coding protocol metrics constituting a fundamental competency requirement. However, the lack of domain-specific expertise in language models constitutes one of the key reasons for their suboptimal performance when addressing tasks within these specialized domains.^8^ Furthermore, the retrieval mechanisms lack capacity to access the implicit conceptual foundations underlying these indicators during relevant text chunk identification processes. Consequently, providing annotations or explanations for every indicator is considered necessary.

During the initial optimization phase, an inconsistent score, *manifesting as score inflation or deflation for the same indicator*, by ChatGPT was observed to exhibit distinct biases across different evaluation metrics, despite using identical prompts and instructions (except for the indicator themselves). Consequently, we try to add independent annotation and scoring criteria for each indicator.

We constructed a reference knowledge base to ensure factual grounding of optimizer-generated annotations and scoring criteria while mitigating terminological inaccuracies in plan evaluation lexicons. This repository systematically aggregates original standardization documents referenced in coding protocol development, discipline-specific academic literature, and professional practice guidelines.

The procedure for adding annotations and scoring criteria is as follows:

(a) Manual Development of Scoring Criteria: Human coders compiled concise three-point scoring criteria (0, 1, 2) with operationalized criteria for each indicator.
b) Initial Annotation Generation: The optimizer was tasked to generate an initial annotation for each indicator based on the knowledge base.
c) Baseline Validation: The validator model conducted evaluation on all plans within the training set, using prompts for each indicator that incorporated its corresponding annotation and the manually edited scoring framework, thereby establishing the minimum preoptimization baseline performance.

#### Optimization of indicator annotations and scoring criteria

The overall optimization procedure is similar to the preceding optimization step:

(a) Prompt Assessment: The refined annotation and scoring criteria are integrated into evaluation prompts, which serve as the validator input. This generates indicator-specific assessment outcomes, subsequently yielding quantitative performance metrics.
(b) Composing Meta-prompt: The meta-prompt for optimization is generated using the indicator, annotation, scoring criteria, corresponding performance, optimization suggestions, and optimization directives.
(c) Prompt Optimization: The meta-prompt is used as input to the optimizer to generate optimized annotation and scoring criteria through knowledge-guided generation.
(d) Iterative Optimization: For indicator-specific annotation and scoring-framework optimization, each indicator was permitted up to seven candidate generations. Candidate retention followed a lexicographic rule: higher accuracy was preferred and, when accuracy was tied, lower MSE was preferred. At an optimization plateau, BZ and YZ assessed whether retained candidates conformed to the human coding standard and contained biased or leading language. A review-approved version could proceed to subsequent use. When a candidate did not pass review, the workflow reverted to the preceding review-approved version. Human review at this stage assessed whether the candidate materials accurately represented the evaluation requirements. The human reference scores remained fixed during this process.

We conducted final plan-quality evaluations on the validation set using GPT-4o and GPT-4o mini with the iteratively optimized prompt framework. During prompt development, we conducted limited sequential comparisons of selected components. On the 10-indicator development subset, Phase 1 optimization increased GPT-4o mini exact-match accuracy from 42% to 56%. On the same subset, unoptimized annotations combined with manually written rubrics achieved 46% exact-match accuracy, Phase 1 optimized prompts achieved 58%, and the Phase 2 optimized framework achieved 78%. Across the five development plans, Phase 2 increased mean exact-match accuracy from 46.78% to 65.35%. These development-stage comparisons examined selected combinations of prompt instructions, annotations, and scoring criteria; they did not estimate the independent effects of individual components or all possible combinations. We also compared performance across GPT-4o and GPT-4o mini under basic and optimized prompt conditions and examined agreement metrics that varied substantially across conditions.

### Quantification and statistical analysis

Most statistical analyses were conducted in Python using custom-written scripts. The archived final-score analysis script requires Python 3.10 or later and uses the Python standard library. During prompt development, candidate prompts were evaluated against human reference scores using exact-match accuracy, with mean squared error (MSE) used to break accuracy ties. Final evaluation comprised 20 held-out validation plan units and 56 indicators, yielding 1,120 planned plan–indicator ratings per model–prompt condition (*n* = 20 plan units; 56 indicators per plan). Exact agreement was calculated as the percentage of available model scores that exactly matched the human reference score. Fifteen of the 4,480 planned ratings did not yield a parseable score (GPT-4o/optimized, *n* = 1; GPT-4o/basic, *n* = 12; GPT-4o mini/basic, *n* = 2; GPT-4o mini/optimized, *n* = 0); these ratings were excluded from primary agreement and class-metric denominators. A sensitivity analysis treated unavailable ratings as disagreements. For the post hoc binary analysis, score 0 was coded as absence (0), and scores 1 and 2 were combined as presence (1).

For each condition, mean plan-level agreement was calculated as the arithmetic mean of the 20 plan-specific agreement percentages. For each model, the optimized-minus-basic difference was calculated within each plan and summarized as the mean paired difference in percentage points. Ninety-five percent confidence intervals (CIs) were estimated from 5,000 nonparametric bootstrap replicates that resampled the 20 validation plan units with replacement (random seed = 20260724); paired differences were resampled at the plan level. Class-specific precision, recall, and F1 scores were calculated for ternary scores 0, 1, and 2 and for the post hoc binary outcomes. Ordinal Krippendorff’s α was calculated for each plan and model–prompt condition using a custom Python script as a complementary measure of model–human agreement. No null-hypothesis tests were conducted. Agreement and CIs are summarized in Tables 3, 4, and 5, class-specific metrics in Table 7, and binary agreement in Table 8.
